# Lipidomic Analyses Reveal Modulation of Lipid Metabolism by the PFAS Perfluoroundecanoic Acid (PFUnDA) in Non-Obese Diabetic Mice

**DOI:** 10.3389/fgene.2021.721507

**Published:** 2021-09-27

**Authors:** Tuulia Hyötyläinen, Johanna Bodin, Daniel Duberg, Hubert Dirven, Unni C. Nygaard, Matej Orešič

**Affiliations:** ^1^ School of Science and Technology, Örebro University, Örebro, Sweden; ^2^ Division of Infection Control and Environmental Health, Norwegian Institute of Public Health, Oslo, Norway; ^3^ School of Medical Sciences, Örebro University, Örebro, Sweden; ^4^ Turku Bioscience Centre, University of Turku and Åbo Akademi University, Turku, Finland

**Keywords:** lipidomics, NOD mice, type 1 diabetes, exposure, PFUnDA

## Abstract

Exposure to Per- and polyfluoroalkyl substances (PFAS) has been linked to multiple undesirable health outcomes across a full lifespan, both in animal models as well as in human epidemiological studies. Immunosuppressive effects of PFAS have been reported, including increased risk of infections and suppressed vaccination responses in early childhood, as well as association with immunotoxicity and diabetes. On a mechanistic level, PFAS exposure has been linked with metabolic disturbances, particularly in lipid metabolism, but the underlying mechanisms are poorly characterized. Herein we explore lipidomic signatures of prenatal and early-life exposure to perfluoroundecanoic acid (PFUnDA) in non-obese diabetic (NOD) mice; an experimental model of autoimmune diabetes. Female NOD mice were exposed to four levels of PFUnDA in drinking water at mating, during gestation and lactation, and during the first weeks of life of female offspring. At offspring age of 11–12 weeks, insulitis and immunological endpoints were assessed, and serum samples were collected for comprehensive lipidomic analyses. We investigated the associations between exposure, lipidomic profile, insulitis grade, number of macrophages and apoptotic, active-caspase-3-positive cells in pancreatic islets. Dose-dependent changes in lipidomic profiles in mice exposed to PFUnDA were observed, with most profound changes seen at the highest exposure levels. Overall, PFUnDA exposure caused downregulation of phospholipids and triacylglycerols containing polyunsaturated fatty acids. Our results show that PFUnDA exposure in NOD mice alters lipid metabolism and is associated with pancreatic insulitis grade. Moreover, the results are in line with those reported in human studies, thus suggesting NOD mice as a suitable model to study the impacts of environmental chemicals on T1D.

## Introduction

Per‐ and polyfluoroalkyl substances (PFAS) are widespread in the environment as they are highly persistent and are used extensively in numerous consumer products and fire-fighting foams. In humans and animals, PFAS are distributed throughout the body *via* circulation, with highest extravascular concentrations being found in the liver and kidneys (Pérez et al., 2013), but with species differences between human and rodents (Fragki et al., 2021). Exposure to PFAS is associated with several undesirable health outcomes throughout life. In animal models, the toxicological effects linked to PFAS exposure include hepatotoxicity, metabolic disturbances including obesity, immunotoxicity, immunosuppression, endocrine disruption, reproductive and developmental toxicity, neurotoxicity, and tumorigenesis (ChristyBarlow et al., 2019). Epidemiological studies in human cohorts have reported similar biological effects associated with PFAS exposure (Lind et al., 2014; Ashley-Martin et al., 2017; Fleisch et al., 2017; Chen et al., 2020; Jin et al., 2020; Sinisalu et al., 2020; Fenton et al., 2021; Schillemans et al., 2021), however, a direct causal connection between the PFAS exposure and specific adverse health outcomes has not yet been identified.

The mechanism of action of PFAS-mediated toxicity has not yet been fully elucidated. However, PFAS exposure has been linked with various metabolic alterations. Several PFAS can activate peroxisome proliferator-activating receptor alpha (PPARα), which is involved in the regulation of lipid metabolism and adipogenesis (Behr et al., 2020). In mammals, there is also data of PPARα-independent transcript regulation, involving activation of the constitutive androstane receptor, estrogen receptor α, liver X receptor, and pregnane X receptor (Rosen et al., 2017). The two most abundant PFAS, namely perfluorooctanoic acid (PFOA) and perfluorooctanesulfonic acid (PFOS), may also affect cholesterol and lipid metabolism through other mechanisms. PFOS and PFOA can disrupt the hepatocyte nuclear factor 4-alpha (HNF4α) signaling pathway, which has a key role in the regulation of lipid homeostasis in hepatocytes (Beggs et al., 2016; Fragki et al., 2021). They can also suppress the cholesterol seven alpha-hydroxylase (CYP7A1) enzyme; the rate-limiting enzyme in bile acid synthesis (Beggs et al., 2016). Moreover, PFAS also interfere with mitochondrial fatty acid β-oxidation in the liver (Lu et al., 2019) and increase the expression of hepatic genes involved in fatty acid and triglyceride synthesis, possibly triggering steatosis through skewing of the balance between lipogenesis and lipolysis (Das et al., 2017). These results are supported by *in vitro* studies in primary human and rat hepatocytes, showing that PFAS exposure results in shift from carbohydrate metabolism to fatty acid accumulation and oxidation (Bjork et al., 2011). Immunotoxic effects of PFOA have also been reported, both *in vitro* and *in vivo* (DeWitt et al., 2009; Corsini et al., 2014), purportedly due to alteration of PPARs, NF-κB regulated gene transactivation and/or regulation of apoptosis (Chain et al., 2020), indicated by inhibition of the T-cell dependent antibody response in animal models.

Epidemiologic studies also report immunosuppressive effects of PFAS, including decreased vaccination responses, increased risk of infections and allergic sensitization in early childhood, immunotoxicity and sex-specific exposure-health associations (Grandjean and Budtz-Jørgensen, 2013; Granum et al., 2013; Predieri et al., 2015; Kvalem et al., 2020). Human studies also link PFOA and PFOS exposure with diabetes incidence, disturbances in glucose metabolism, and insulin secretion (Conway et al., 2016; Domazet et al., 2016). We have previously identified associations between prenatal exposure to PFAS exposure and two autoimmune diseases, namely, type 1 diabetes (T1D) and celiac disease (CD) (McGlinchey et al., 2019; Sinisalu et al., 2020). Understanding of the mechanism through which PFAS contribute to the pathogenesis and increased risk of T1D is currently lacking, although roles for impaired beta/immune-cell function and immunomodulation have been suggested (Bodin et al., 2015a). Notably, PFOA and PFOS may disrupt production of human pancreatic progenitor cells (Liu et al., 2018), thus suggesting that these two compounds could disturb the formation of the mature pancreas, with clear implications for the risk of T1D (Liu et al., 2018). In a non-obese diabetic (NOD) mice, an experimental model of autoimmune diabetes, prenatal and early-life exposure to perfluoroundecanoic acid (PFUnDA) was shown to exacerbate insulitis development, i.e., inflammation in the pancreas which is a prerequisite for diabetes development (Bodin et al., 2016). The study also showed that exposure to PFUnDA caused an increase in the number of apoptotic pancreatic islet cells prior to insulitis, along with reduced phagocytotic activity of peritoneal macrophages (Bodin et al., 2016). The latter is one of the hallmarks of progression to autoimmune diabetes in NOD mice (O’Brien et al., 2002).

In the present work we study the impact of exposure to PFUnDA on lipidomic profiles in NOD mice, and link this with previously-reported early markers for autoimmune diabetes (Bodin et al., 2016): 1) insulitis grade, 2) number of macrophages (MP) and 3) apoptotic, active caspase-3 positive cells (Aac3P) in pancreatic islets. PFUnDA was chosen as an exemplar PFAS as, in *in vitro* systems, it has been shown to disturb macrophage function. Moreover, our human studies have shown that PFUnDA in particular, in addition to perfluorononanoic acid (PFNA), is strongly associated with changes in the lipidome (Salihovic et al., 2018). Further, we compared our results with our previous human studies to assess the applicability of the NOD mouse model for exposure assessment and translation of the results into human studies.

## Methods

### NOD Mouse Study

The study set-up of the NOD mouse study was as reported previously (Bodin et al., 2016). Briefly, NOD/ShiLtJ mice from the Jackson Laboratory (Maine, United States) were used for breeding at 8 and 10 weeks of age and randomly allocated to the exposure groups. Female offspring were exposed to PFUnDA from the time of parental mating, through their gestation and early life until 11–12 weeks of age when the serum samples were collected, with 4–5 mice kept per cage and 8 mice per exposure group.

Exposure was through drinking water. The four exposure groups included: 1) negative control (autoclaved water only), 2) PFUnDA 3 μg/ml (CAS: 2058-94-8, >96% purity, Santa Cruz Biotechnology, Dallas, United States), 3) PFUnDA 30 μg/ml and 4) PFUnDA 300 μg/ml. The exposure doses correspond to ∼0.417, 4.17 and 41.7 μg/kg bw/day (calculated based on mean mouse weight of 23 g (all mice had similar body weights throughout the treatment period) and mean measured volume of drinking water consumption of 3.2 ml/day at 10 weeks of age).

All experiments were performed conforming to the laws and regulations for experiments on live animals in Norway and were approved by the local representative of the Norwegian Animal Research Authority. In the NOD mouse model, insulitis is the most prominent feature preceding diabetes onset, with impaired macrophage phagocytosis being associated with seroconversion. Insulitis was assessed by grading of hematoxylin and eosin-stained pancreatic tissue sections. Early signs of insulitis include an increased number of apoptotic cells, decreased number of tissue-resident macrophages in pancreatic islets and reduced phagocytic function of macrophages isolated from the peritoneum. The animals’ characteristics after exposure are summarized in Table 1 as mean number of insulitis grades, identified tissue resident macrophages and apoptotic cells in pancreatic islets from mice at 7 and 11 weeks of age (Bodin et al., 2016).

**TABLE 1:** NOD mouse histological characteristics at 7 and 11 weeks of age after lifelong PFUnDA exposure, represented as overall insulitis grade and the mean numbers of macrophages and apoptotic cells in pancreatic islets prior to insulitis (with insulitis grade 0). Mean of eight female NOD mice/group.

Exposure level (µg/ml drinking water)	Insulitis grade, 7 weeks	Insulitis grade, 11 weeks	Number of macrophages, 7 weeks	Number of macrophages, 11 weeks	Number of apoptotic cells, 7 weeks	Number of apoptotic cells, 11 weeks
**0**	1.63	2.30	2.25	7.00	0.62	0.68
**3**	1.28	2.23	1.75	1.60	0.10	0.77
**30**	2.00	2.50	1.75	4.55	0.55	0.88
**300**	2.20	3.35	0.50	4.00	0.95	2.42

### Determination of Insulitis Grade and Cell Counts

Histological evaluation and cell counts were performed as reported previously (Bodin et al., 2016). For histological evaluation, the pancreas was fixed in formalin, embedded in paraffin and processed as previously described prior to hematoxylin and eosin staining (Bodin et al., 2013). Insulitis was assessed as the area of an islet infiltrated by lymphocytes. 0% infiltration = grade 0, periinsulitis and up to 10% infiltration = grade 1, 10–49% infiltration = grade 2, 50–74% infiltration = grade 3 and 75–100% infiltration = grade 4, as illustrated previously (Bodin et al., 2013). The final insulitis grade per mouse was determined as the mean insulitis grade from six pancreatic sections, for a total of 60–75 islets.

**FIGURE 1:**
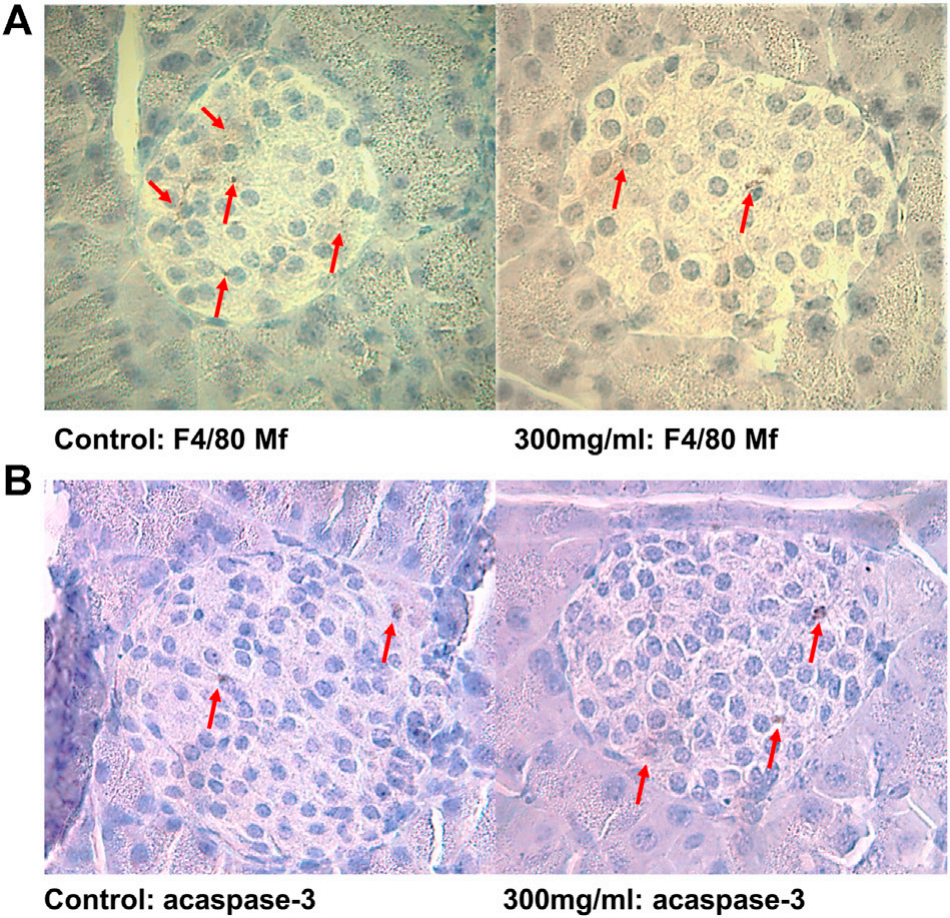
**(A)** Representative pancreatic islets immunostained for tissue-resident F4/80 positive macrophages and **(B)** apoptotic active caspase-3 positive cells from control (0 μg/ml) and PFUnDA 300 μg/ml exposed mice, respectively. Modified from ref. (Bodin et al., 2016). Scale bar indicates 40 μm.

Formalin-fixed pancreatic sections were stained overnight with antibodies against F4/80 (tissue resident macrophages, AbD Serotec, Oxford, United Kingdom, 1:50) and active caspase-3 (apoptotic cells, Cell Signalling Technology, Beverly, MA, United States, 1:400), as previously described (Bodin et al., 2013). Since most female NOD mice spontaneously develop insulitis with age, the pre-insulitis islets are expected to give most information on early accelerating effects, thus the number of positive cells from islets with grade 0 are presented for each antibody staining. A representative figure is shown in Figures 1A,B.

Peritoneal macrophages were from mice at 7 and 11 weeks (n = 8 for both time points) by peritoneal lavage with, in total, 6 ml of PBS as previously described (Bodin et al., 2015b). The cell suspension was centrifuged at 250 x *g* for 10 min and erythrocytes were removed by dissolving the cell pellet in 0.2% NaCl on ice for 4 min. Cells were seeded at 2 × 10^6^ cells/ml in 48 wells plates with RPMI cell culture medium (Biowest, Nuaillé, France) and, after 1 h in culture, 85–95% of the attached cells are assumed to be macrophages. The attached cells were incubated in fresh medium overnight. To analyse the phagocytic function, FITC-labelled Zymosan particles were added at a ratio of 20 particles per cell (Invitrogen Life Technologies, Carlsbad, California, United States) and incubated for 30 min at 37°C. The cells were washed twice in PBS and incubated with accutase (In vitrogen Life Technologies) for at least 15 min to remove particles from the cell surfaces and to detach cells. Thereafter, the cells were washed with PBS and fixed in 0.2% paraformaldehyde in RPMI 1640 phenol red-free cell culture medium (Biowest) prior to assessment of phagocytic activity (FITC intensity per cell) by flow cytometry (LSR II, BD Bioscience, Franklin Lakes, NJ, United States).

### Lipidomics

Serum samples collected at necropsy (11 weeks) were randomized and extracted using a modified version of the previously-published Folch procedure (Heli Nygren and Castillo, 2011). In short, 10 µl of 0.9% NaCl and, 120 µl of CHCl3: MeOH (2:1, v/v) containing the internal standards (c = 2.5 μg/ml) was added to 10 µl of each serum sample. The standard solution contained the following compounds: 1,2-diheptadecanoyl-sn-glycero-3-phosphoethanolamine [PE(17:0/17:0)], N-heptadecanoyl-D-erythro-sphingosylphosphorylcholine [SM(d18:1/17:0)], N-heptadecanoyl-D-erythro-sphingosine [Cer(d18:1/17:0)], 1,2-diheptadecanoyl-sn-glycero-3-phosphocholine [PC(17:0/17:0)], 1-heptadecanoyl-2-hydroxy-sn-glycero-3-phosphocholine [LPC(17:0)] and 1-palmitoyl-d31-2-oleoyl-sn-glycero-3-phosphocholine [PC(16:0/d31/18:1)], were purchased from Avanti Polar Lipids, Inc (Alabaster, AL, United States), and, triheptadecanoylglycerol [TG(17:0/17:0/17:0)] was purchased from Larodan AB (Solna, Sweden). The samples were vortex mixed, incubated on ice for 30 min and then were centrifuged (9,400 × *g*, 3 min). 60 µL from the lower layer was then collected and diluted with 60 µL of CHCl3: MeOH (2:1, v/v). The samples were stored at −80°C until analysis.

Calibration curves using 1-hexadecyl-2-(9Z-octadecenoyl)-sn-glycero-3-phosphocholine [PC(16:0e/18:1(9Z))], 1-(1Z-octadecenyl)-2-(9Z-octadecenoyl)-sn-glycero-3-phosphocholine [PC(18:0p/18:1(9Z))], 1-stearoyl-2-hydroxy-sn-glycero-3-phosphocholine [LPC(18:0)], 1-oleoyl-2-hydroxy-sn-glycero-3-phosphocholine [LPC(18:1)], 1-palmitoyl-2-oleoyl-sn-glycero-3-phosphoethanolamine [PE(16:0/18:1)], 1-(1Z-octadecenyl)-2-docosahexaenoyl-sn-glycero-3-phosphocholine [PC(18:0p/22:6)] and 1-stearoyl-2-linoleoyl-sn-glycerol [DG(18:0/18:2)], 1-(9Z-octadecenoyl)-sn-glycero-3-phosphoethanolamine [LPE(18:1)], N-(9Z-octadecenoyl)-sphinganine [Cer(d18:0/18:1(9Z))], 1-hexadecyl-2-(9Z-octadecenoyl)-sn-glycero-3-phosphoethanolamine [PE(16:0/18:1)] from Avanti Polar Lipids, 1-Palmitoyl-2-Hydroxy-sn-Glycero-3-Phosphatidylcholine [LPC(16:0)], 1,2,3 trihexadecanoalglycerol [TG(16:0/16:0/16:0)], 1,2,3-trioctadecanoylglycerol [TG(18:0/18:0/18)] and 3β-hydroxy-5-cholestene-3-stearate [ChoE(18:0)], 3β-Hydroxy-5-cholestene-3-linoleate [ChoE(18:2)] from Larodan, were prepared to the following concentration levels: 100, 500, 1,000, 1,500, 2000 and 2,500 ng/ml (in CHCl3:MeOH, 2:1, v/v) including 1,250 ng/ml of each internal standard. The calibration curves are specified in Supplementary Table S1.

The samples were analyzed by ultra-high-performance liquid chromatography quadrupole time-of-flight mass spectrometry (UHPLC-QTOFMS). Briefly, the UHPLC system used in this work was a 1,290 Infinity II system from Agilent Technologies (Santa Clara, CA, United States). The system was equipped with a multi sampler (maintained at 10°C), a quaternary solvent manager and a column thermostat (maintained at 50°C). Injection volume was 1 µl and the separations were performed on an ACQUITY UPLC^®^ BEH C18 column (2.1 mm × 100 mm, particle size 1.7 µm) by Waters (Milford, MA, United States). The mass spectrometer coupled to the UHPLC was a 6545 QTOF from Agilent Technologies interfaced with a dual jet stream electrospray (Dual ESI) ion source. All analyses were performed in positive ion mode and MassHunter B.06.01 (Agilent Technologies) was used for all data acquisition. Quality control was performed throughout the dataset by including blanks, pure standard samples, extracted standard samples and control serum samples (Pluskal et al., 2010). The lipid concentrations in pooled control samples showed % RSDs within accepted analytical limits at average of 7.3% for serum samples (Supplementary Table S2).

Mass spectrometry data processing was performed using the open source software package MZmine 2.18 (Pluskal et al., 2010). The following steps were applied in this processing: 1) Crop filtering with a m/z range of 350–1,200 m/z and an RT range of 2.0–12 min, 2) Mass detection with a noise level of 750, 3) Chromatogram builder with a minimum time span of 0.08 min, minimum height of 1,000 and a m/z tolerance of 0.006 m/z or 10.0 ppm, 4) Chromatogram deconvolution using the local minimum search algorithm with a 70% chromatographic threshold, 0.05 min minimum RT range, 5% minimum relative height, 1,200 minimum absolute height, a minimum ration of peak top/edge of 1.2 and a peak duration range of 0.08–5.0, 5), Isotopic peak grouper with a m/z tolerance of 5.0 ppm, RT tolerance of 0.05 min, maximum charge of two and with the most intense isotope set as the representative isotope, 6) Peak filter with minimum 12 data points, a FWHM between 0.0 and 0.2, tailing factor between 0.45 and 2.22 and asymmetry factor between 0.40 and 2.50, 7) Join aligner with a m/z tolerance of 0.009 or 10.0 ppm and a weight for of 2, a RT tolerance of 0.1 min and a weight of one and with no requirement of charge state or ID and no comparison of isotope pattern 8) Peak list row filter with a minimum of 10% of the samples 9) Gap filling using the same RT and m/z range gap filler algorithm with an m/z tolerance of 0.009 m/z or 11.0 ppm, 10) Identification of lipids using a custom database search with an m/z tolerance of 0.009 m/z or 10.0 ppm and a RT tolerance of 0.15 min, and 11) Normalization was done by using internal standards PE (17:0/17:0), SM (d18:1/17:0), Cer (d18:1/17:0), LPC (17:0), TG (17:0/17:0/17:0) and PC [16:0/d30/18:1)] followed by calculation of the concentrations based on lipid-class concentration curves.

### Statistical Analysis

The data were log2 transformed and autoscaled, i.e., normalized to zero mean and unit variance prior the statistical analyses. The statistical analyses were performed using MetaboAnalyst 4 (Chong et al., 2019), MeV and MetScape three for CytoScape (Basu et al., 2017). The impact of PFUnDA levels on the lipidome was investigated using analysis of variance (ANOVA), using Fisher’s Least Significant Difference (LSD) as *post-hoc* analysis. Additionally, two-sample t-tests with Fisher’s LSD as *post-hoc* analysis was used to compare the highest exposure group to the control group. The Debiased Sparse Partial Correlation (DSPC) algorithm was used for estimating partial correlation networks, visualized by MetScape4 with cut-off values off correlations between ± 0.25 to 1.0. DSPC was used to distinguish difference between direct and indirect associations and reconstruct a network that calculates partial correlation coefficients and P-values for every metabolic feature pair (Chong et al., 2019). A Spearman’s rank correlation analysis was used assess correlation between PFUnDA, insulitis grade and the lipids. Significance level was set at P < 0.05.

## Results

The 32 female NOD mice were divided into four different exposure groups (exposed to 0, 3, 30 and 300 μg/ml of PFUnDA). In serum samples, we identified 170 serum lipids from nine major lipid classes including triacylglycerols (TG), ceramides (Cer), cholesterol esters (CE), diacylglycerols (DG), lysophosphatidylcholines (LPC), phosphatidylcholines (PC) [including subclass of ether PCs (O-PC)], phosphatidylethanolamines (PE), phosphatidylinositols (PI), and sphingomyelins (SM) (Supplementary Figure S1).

### Exposure Caused Dose-dependent Changes in Lipid Levels

**FIGURE 2:**
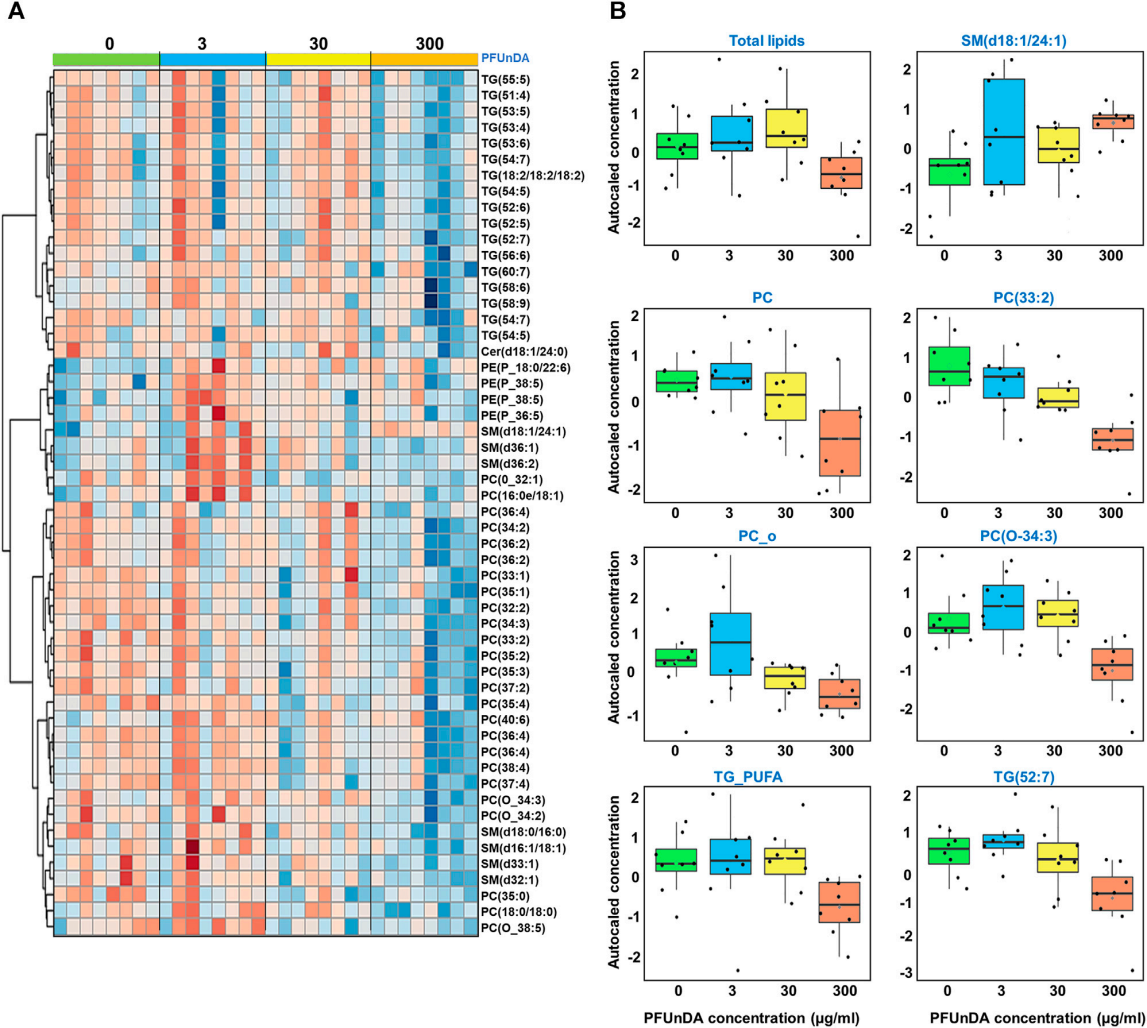
**(A)** Heatmap showing levels of lipids that are significantly different across the exposure groups and **(B)** selected examples covering lipid groups and selected individual lipids showing significant differences in ANOVA (FDR *p* < 0.05).

We first applied ANOVA analysis to investigate the impact of PFUnDA exposure on circulating lipid levels (at 11 weeks of age), both in the levels of lipid classes and in individual lipid level (Table 2; Figures 2A,B). The strongest, significant changes observed occurred in mice treated with the highest dose (ctrl *vs* 300 μg/ml), with significantly-decreased levels of 47 lipids (17 PC, 2 O-PC, 3 SM and 10 TG), with only one SM [SM(d18:1/24:1)] increased. At lower exposure levels, changes in lipid levels did not reach statistical significance after correction for False Discovery Rate (FDR). The TGs showing significant differences were those specific TGs that contained polyunsaturated fatty acyls (PUFAs, sum of double bonds 4–7).

**TABLE 2:** Lipids showing significant differences between the exposure levels, ANOVA analysis, as well as fold changes (FCs) between the control and the highest exposure group, with nominal and FDR-corrected *p*-values.

Lipid	f	p	FDR	Fisher’s LSD	FC (high vs. ctrl)	p (300 vs. ctrl)	FDR p
PC(33:2)	10.607	7.88E-05	0.014	0–3; 0–30; 0–300; 3–30; 3–300; 30–300	0.59	2.08E-04	0.013
PC(32:2)	9.6644	1.52E-04	0.014	0–3; 0–30; 0–300; 3–30; 3–300; 30–300	0.54	3.22E-06	0.001
PC(O-34:3)	7.2902	9.24E-04	0.042	3–0; 30–0; 0–300; 3–30; 3–300; 30–300	0.73	4.45E-03	0.049
PC(O-34:2)	6.6341	1.59E-03	0.042	0–3; 0–30; 0–300; 3–30; 3–300; 30–300	0.72	1.71E-03	0.039
PC(35:2)	6.5107	1.76E-03	0.042	0–3; 0–30; 0–300; 3–30; 3–300; 30–300	0.69	1.21E-03	0.036
PC(O-32:1)	6.4613	1.84E-03	0.042	3–0; 0–30; 0–300; 3–30; 3–300; 30–300	0.85	1.85E-02	0.068
PC(34:2)	6.3803	1.97E-03	0.042	0–3; 0–30; 0–300; 3–30; 3–300; 30–300	0.82	1.38E-03	0.036
PC(35:4)	6.3684	1.99E-03	0.042	0–3; 0–30; 0–300; 3–30; 3–300; 30–300	0.65	6.42E-03	0.049
PC(36:2)	6.304	2.10E-03	0.042	0–3; 0–30; 0–300; 3–30; 3–300; 30–300	0.83	6.79E-04	0.031
TG(52:7)	5.9586	2.83E-03	0.045	3–0; 0–30; 0–300; 3–30; 3–300; 30–300	0.50	8.16E-03	0.049
PC(18:0/18:0)	5.9074	2.96E-03	0.045	3–0; 30–0; 0–300; 3–30; 3–300; 30–300	0.85	6.66E-03	0.049
PC 36:4	5.8302	3.16E-03	0.045	3–0; 0–30; 0–300; 3–30; 3–300; 30–300	0.66	4.66E-03	0.049
PC(35:0)	5.7934	3.27E-03	0.045	0–3; 0–30; 0–300; 3–30; 3–300; 30–300	0.81	9.69E-04	0.035
PC(36:2)	5.7182	3.49E-03	0.045	0–3; 0–30; 0–300; 3–30; 3–300; 30–300	0.84	3.09E-03	0.045
PC(38:4)	5.5776	3.96E-03	0.048	3–0; 0–30; 0–300; 3–30; 3–300; 30–300	0.77	6.24E-03	0.049
TG(60:7)	5.3521	4.84E-03	0.055	3–0; 30–0; 0–300; 3–30; 3–300; 30–300	0.58	2.98E-02	0.093
PC(37:4)	5.0547	6.35E-03	0.064	0–3; 0–30; 0–300; 3–30; 3–300; 30–300	0.69	8.47E-03	0.049
SM(d18:0/16:0)	5.0537	6.36E-03	0.064	3–0; 0–30; 0–300; 3–30; 3–300; 30–300	0.78	2.71E-03	0.045
SM(d33:1)	4.8431	7.73E-03	0.070	3–0; 0–30; 0–300; 3–30; 3–300; 30–300	0.83	3.42E-03	0.045
PC(O-38:5)	4.8409	7.74E-03	0.070	3–0; 0–30; 0–300; 3–30; 3–300; 30–300	0.83	2.41E-02	0.085
SM(d34:2)	4.7925	8.10E-03	0.070	3–0; 0–30; 0–300; 3–30; 3–300; 30–300	0.87	1.06E-02	0.052
SM(d32:1)	4.69	8.91E-03	0.071	0–3; 0–30; 0–300; 3–30; 3–300; 30–300	0.81	4.06E-03	0.049
TG(54:5)	4.6519	9.24E-03	0.071	0–3; 30–0; 0–300; 30–3; 3–300; 30–300	0.35	1.70E-02	0.067
TG(53:6)	4.6278	9.45E-03	0.071	0–3; 30–0; 0–300; 30–3; 3–300; 30–300	0.43	7.31E-03	0.049
PC(34:3)	4.4571	1.11E-02	0.077	0–3; 0–30; 0–300; 3–30; 3–300; 30–300	0.71	1.97E-03	0.039
PC(36:4)	4.4213	1.15E-02	0.077	3–0; 0–30; 0–300; 3–30; 3–300; 30–300	0.78	1.68E-02	0.067
Cer(d18:1/24:0)	4.3688	1.21E-02	0.078	0–3; 0–30; 0–300; 30–3; 3–300; 30–300	0.76	6.04E-03	0.049
PC(O-38:5)	4.2951	1.30E-02	0.079	3–0; 30–0; 300–0; 3–30; 3–300; 300–30	1.09	2.04E-01	0.298
SM(d36:1)	4.2905	1.30E-02	0.079	3–0; 30–0; 0–300; 3–30; 3–300; 30–300	0.88	1.04E-01	0.202
TG(54:7)	4.1007	1.57E-02	0.087	0–3; 0–30; 0–300; 30–3; 3–300; 30–300	0.37	1.44E-02	0.062
PC(35:3)	4.0899	1.58E-02	0.087	0–3; 0–30; 0–300; 3–30; 3–300; 30–300	0.69	3.45E-03	0.045
TG(52:6)	4.0459	1.65E-02	0.088	3–0; 30–0; 0–300; 30–3; 3–300; 30–300	0.60	8.69E-03	0.049
PC(33:1)	3.8048	2.09E-02	0.100	0–3; 0–30; 0–300; 30–3; 3–300; 30–300	0.69	1.98E-04	0.013
SM(d18:1/24:1)	3.1579	4.02E-02	0.135	3–0; 30–0; 300–0; 3–30; 300–3; 300–30	1.20	2.17E-03	0.039
PC(35:1)	3.6728	2.39E-02	0.108	0–3; 0–30; 0–300; 3–30; 3–300; 30–300	0.79	6.02E-03	0.049
TG(54:5)	3.595	2.58E-02	0.111	0–3; 30–0; 0–300; 30–3; 3–300; 30–300	0.66	6.53E-03	0.049
TG(53:5)	3.3043	3.46E-02	0.128	0–3; 0–30; 0–300; 30–3; 3–300; 30–300	0.62	6.82E-03	0.049
TG(53:4)	3.0307	4.58E-02	0.148	3–0; 0–30; 0–300; 3–30; 3–300; 30–300	0.64	7.19E-03	0.049
TG(55:5)	3.204	3.83E-02	0.132	3–0; 0–30; 0–300; 3–30; 3–300; 30–300	0.63	7.94E-03	0.049
TG(51:4)	3.2689	3.59E-02	0.130	0–3; 30–0; 0–300; 30–3; 3–300; 30–300	0.61	8.23E-03	0.049
PC(37:2)	3.7714	2.16E-02	0.100	0–3; 0–30; 0–300; 3–30; 3–300; 30–300	0.73	9.01E-03	0.049
TG(52:5)	3.2148	3.79E-02	0.132	0–3; 30–0; 0–300; 30–3; 3–300; 30–300	0.63	9.39E-03	0.050
TG(53:3)	2.0444	1.30E-01	0.266	3–0; 30–0; 0–300; 30–3; 3–300; 30–300	0.69	9.64E-03	0.050
TG(18:2/18:1/18:1)	2.0095	1.35E-01	0.269	0–3; 0–30; 0–300; 30–3; 3–300; 30–300	0.76	1.13E-02	0.054
TG(54:4)	2.3716	9.17E-02	0.218	0–3; 0–30; 0–300; 3–30; 3–300; 30–300	0.73	1.16E-02	0.054
TG(18:1/18:2/18:2)	2.6566	6.77E-02	0.180	0–3; 30–0; 0–300; 30–3; 3–300; 30–300	0.71	1.22E-02	0.055
TG(54:7)	3.6252	2.50E-02	0.111	0–3; 30–0; 0–300; 30–3; 3–300; 30–300	0.48	1.34E-02	0.059
TG(48:3)	1.901	1.52E-01	0.290	0–3; 30–0; 0–300; 30–3; 3–300; 30–300	0.74	1.49E-02	0.063
TG(50:5)	2.2448	1.05E-01	0.244	0–3; 30–0; 0–300; 30–3; 3–300; 30–300	0.67	1.54E-02	0.064
TG(18:2/18:2/18:2)	3.1965	3.86E-02	0.132	0–3; 30–0; 0–300; 30–3; 3–300; 30–300	0.62	1.79E-02	0.068
TG(16:0/18:2/18:3)	2.6964	6.50E-02	0.175	0–3; 30–0; 0–300; 30–3; 3–300; 30–300	0.66	1.85E-02	0.068
Cer(d18:1/23:0)	2.5799	7.35E-02	0.190	0–3; 0–30; 0–300; 30–3; 3–300; 30–300	0.78	2.30E-02	0.083
TG(54:6)	2.3473	9.41E-02	0.221	0–3; 30–0; 0–300; 30–3; 3–300; 30–300	0.60	2.43E-02	0.085
TG(51:3)	1.6392	2.03E-01	0.328	0–3; 30–0; 0–300; 30–3; 3–300; 30–300	0.71	2.52E-02	0.086
TG(52:4)	2.8753	5.39E-02	0.161	3–0; 30–0; 0–300; 30–3; 3–300; 30–300	0.74	2.63E-02	0.088
TG(16:0/18:2/18:2)	2.7629	6.06E-02	0.174	3–0; 30–0; 0–300; 30–3; 3–300; 30–300	0.76	2.77E-02	0.090
TG(49:3)	2.0405	1.31E-01	0.266	0–3; 30–0; 0–300; 30–3; 3–300; 30–300	0.67	2.97E-02	0.093

**FIGURE 3:**
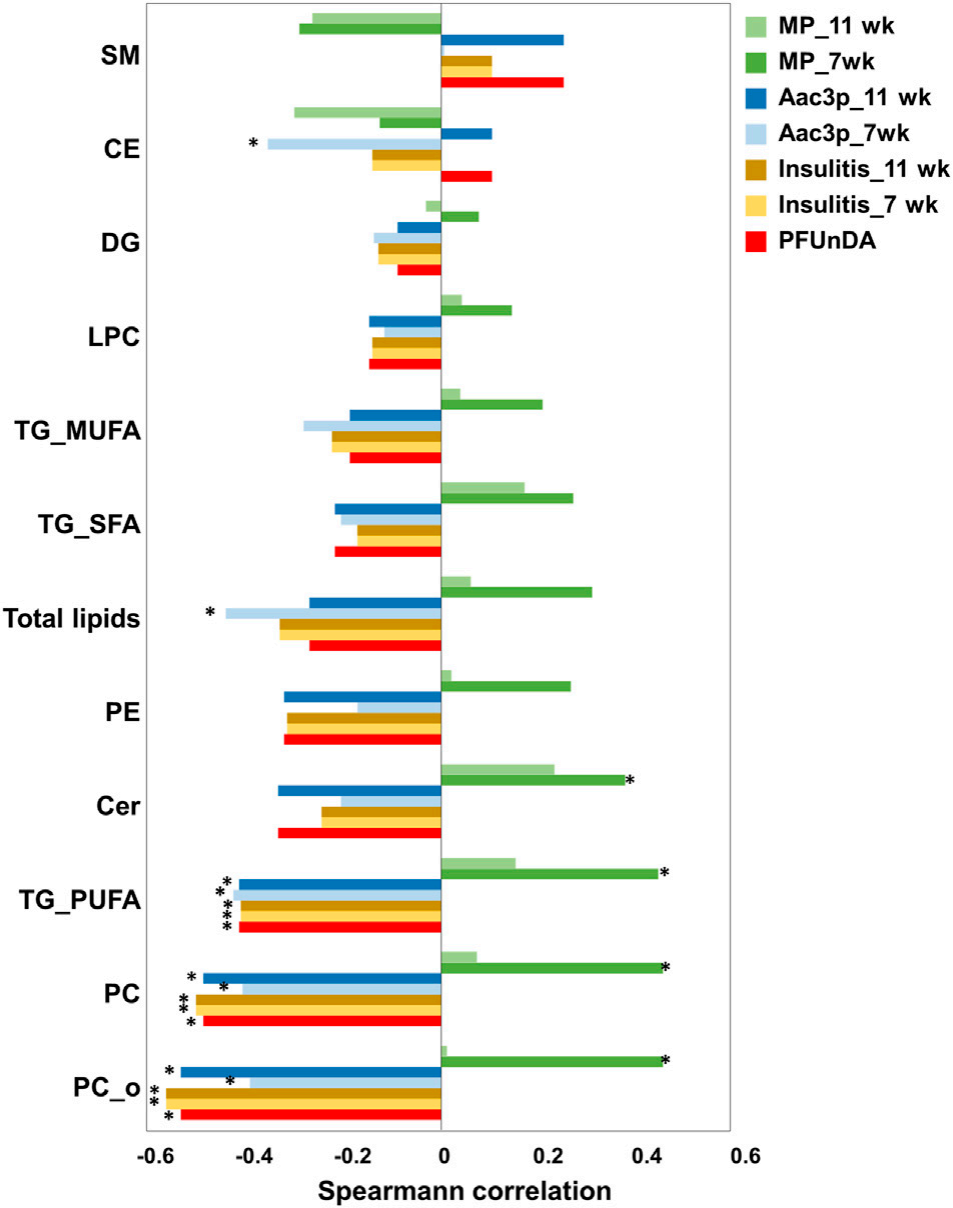
Spearman correlation between lipid classes and PFUnDA exposure levels, insulitis grade, number of macrophages (MPs) and number of Aac3P in pancreatic islets. *p < 0.05.

Next, we investigated the dose-response characteristics between PFUnDA exposure and lipids, using Spearman’s rank correlation, both at the lipid class level and at the level of individual lipids (Figure 3; Table 2). Statistically significant inverse correlations between exposure level and lipid classes were observed in PC, O-PC and TG_PUFA classes, while the other lipid classes showed similar negative trends, with CE and SM having an opposite trend. A total of 29 individual lipids (17 PC, 5 O-PC, 4 SM, 2TGs and 1 Cer) showed negative correlations and one SM showed a positive correlation with the PFUnDA levels.

**FIGURE 4:**
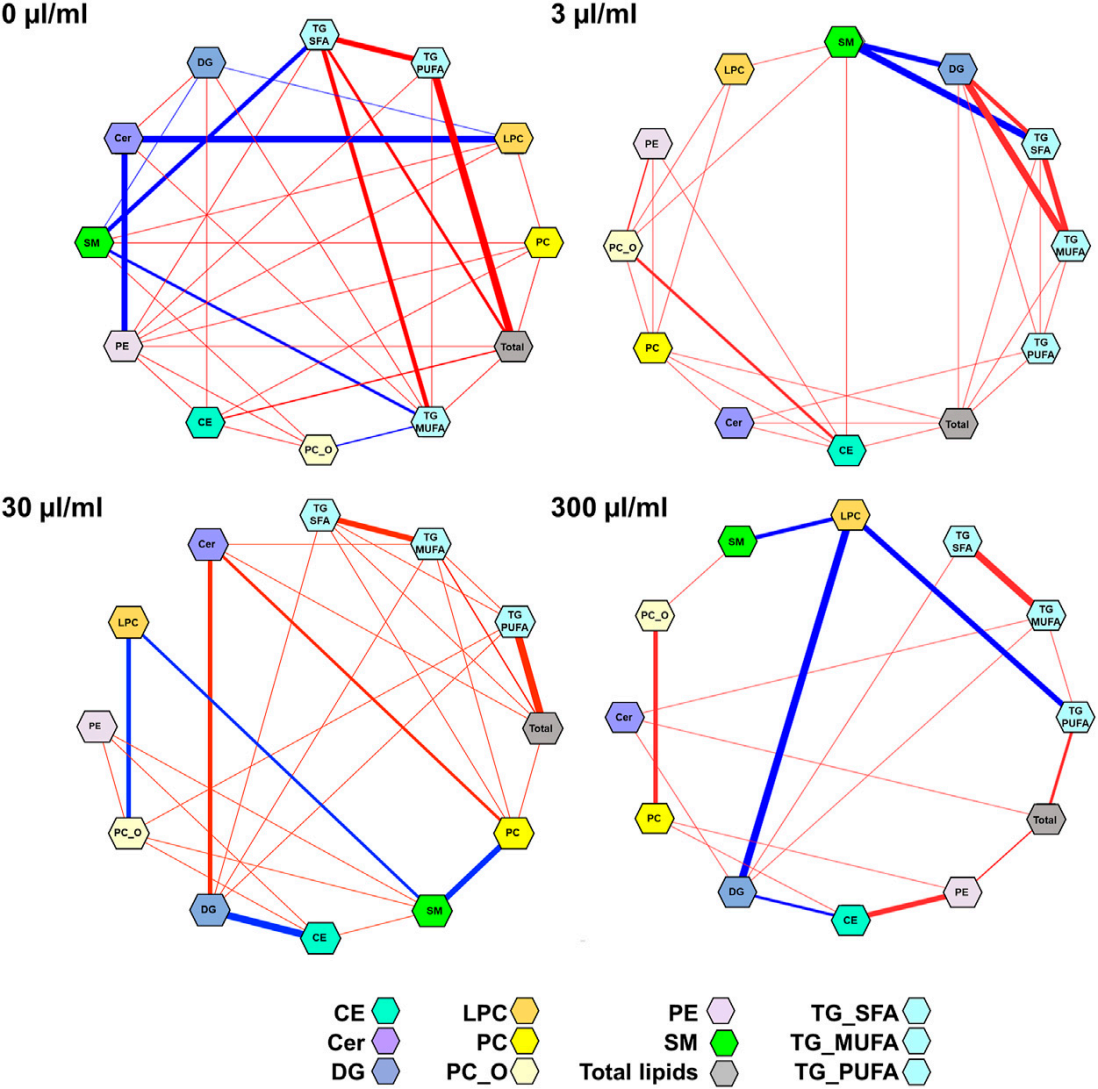
Partial correlation network of lipid classes at different PFUnDA exposure levels (0, 3, 30 and 300 μg/ml in drinking water). Here, each node represents a lipid class(sum of lipids, after autoscaling for each lipid class) in, and each edge represents the strength of partial correlation between two parameters. Edge weights represent the partial correlation coefficients, with Edge colors: blue color for negative correlations and red for positive correlations, the thickness of the line shows the strength of the correlation. Edge ranges adjusted between ±0.25 and 1.0.

We then applied DSPC analysis to investigate the different lipid-lipid associations across different exposure levels (Figure 4). The associations (edges in the networks) between the lipid classes decreased with the increasing PFUnDA exposure levels.

### Altered Lipid Levels Associated With Insulitis Grade, Number of Tissue Resident Macrophages and Apoptotic, Active Caspase-3 Positive Cells in Pancreatic Islets

**FIGURE 5:**
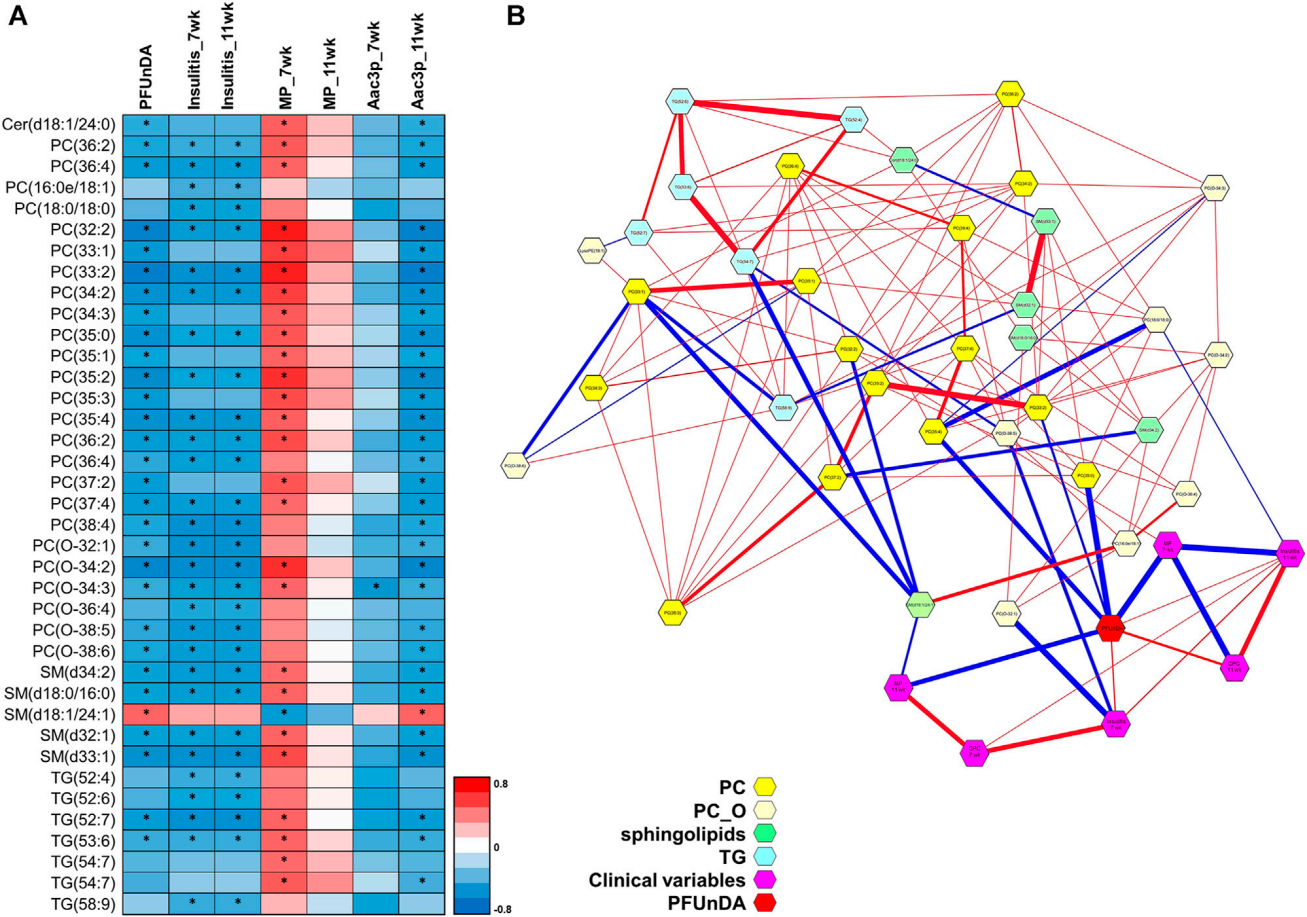
**(A)** Individual lipids showing significant Spearman correlation and PFUnDA exposure levels, insulitis grade, number of macrophages (MPs) and number of apoptotic, active caspase-3 positive cells in pancreatic islets. Significant changes marked with *. **(B)** Partial correlation network. Here, each node represents a compound or a clinical parameter, and edges represent the strength of partial correlation between two compounds/parameters. Edge thicknesses represent the strength of the partial correlation coefficients. Edge colors: blue color for negative correlations and red for positive correlations. Edge ranges adjusted between ±0.25 and 1.0.

Specific lipid classes (Figure 3) as well as individual lipids (Figure 5A) correlated with insulitis grade at 7 and 11 weeks of age. The lipid classes that correlated with PFUnDA exposure levels were also inversely associated with insulitis grade, i.e., PC, O-PC and TG_PUFA (Figure 3). A total of 29 lipids were inversely associated (13PC, 7 O-PC, 4 SM and 5TG) with insulitis grade.

Lipid levels also associated with the number of tissue resident macrophages (MPs) in pancreatic islets prior to insulitis (in islets with insulitis grade 0), particularly with MPs at week 7 (Figures 3, 5A). The same lipid classes that were found inversely associated with PFUnDA and insulitis grade, were also positively associated with MPs. Additionally, ceramides were also positively correlated with the number of MPs. Altogether, 31 lipids were either positively or negatively associated (FDR-adjusted P < 0.05) with the number of MPs at week 7 (17 PC, 5 O-P, 5 SM, 4TGs, 1 Cer), while the associations at week 11 showed similar trends, however, did not reaching statistical significance. Also, two other ceramides showed a trend of positive correlation with MPs at week 7 (R> 0.35, nominal P < 0.05, FDR-corrected P = 0.08–0.13). There were also significant associations between the same lipid classes as for PFUnDA and insulitis grade) at week 7 and 11, and additionally an inverse association with the total lipids and CEs at week 7. At the level of individual lipids, one lipid [O-PC(34:3)] at week 7 and 30 lipids at week 11 (17 PC, 5 O-PC, 4 SM, 3 TG, 1 Cer) showed negative association with the number of apoptotic, active caspase-3 positive cells (Aac3P) in pancreatic islets. Additionally, at week 11, one sphingolipid showed a positive association. The direction of associations was opposite between the macrophages and lipids when compared the associations between and active caspase-3 positive cells and lipids. Moreover, the associations were significant only at the earlier time point of 7 weeks.

By using DSPC analysis, we then further investigated the associations between lipids, PFUnDA, insulitis grade, and the number of tissue-resident macrophages and apoptotic, active caspase-3 positive cells in pancreatic islets (Figure 5B). The PFUnDA exposure levels, insulitis grade and the number of MPs and Aac3P s were closely associated with each other, as previously reported (Bodin et al., 2016), and further with multiple lipids.

## Discussion

In NOD mice exposed to PFUnDA during early life, we observed dose-dependent changes in lipidomic profiles, as well as disruption of the lipid-lipid correlation network. The network analysis indicated that PFUnDA exposure decreased the normally tightly-controlled process of lipid homeostasis and strong intralipid class correlations in a dose-dependent manner. The largest changes were seen at the highest exposure levels, the effect being clearly dose-dependent. As we have demonstrated earlier, the low and medium exposure levels seemed to have even a protective effect on the development of T1D, as evaluated by the indicators of the diabetes development (insulitis, number of macrophages) however, the disrupted lipid-lipid interactions showed that there are metabolite level changes even at lower exposure levels. Overall, PFUnDA exposure caused downregulation of phospholipids and PUFA-containing TGs in the circulation. It has been hypothesized that PUFA containing TGs are functionally involved in apoptosis (Al-Saffar et al., 2002; Li et al., 2018). These lipids have been shown to accumulate in cells during apoptosis, potentially protecting cells from lipid peroxide-induced membrane damage under increased levels of oxidative stress (Li et al., 2018). Thus, depletion of PUFA containing TGs in circulation, as found in our current study, could be due to their accumulation into the apoptotic cells (Bi et al., 2017).

Importantly, the levels of several lipids were also associated with insulitis grade, as well as with the number of MPs and Aac3P cells. The presence of these cells in the pancreas are linked with the development of T1D, as macrophages are key to the autoimmune-mediated destruction of β cells (O’Brien et al., 2002; Bosurgi et al., 2017), while apoptotic cells stimulate a tissue repair program in macrophages (Bosurgi et al., 2017). As reported in our previous study, PFUnDA exposure was associated with accelerated insulitis development, which was further shown to be linked with a decreased number and/or function of tissue-resident macrophages in pancreatic islets prior to insulitis (Bodin et al., 2016). The latter may then lead to an increased number of apoptotic cells and autoantigens required in the activation of an autoimmune response. However, as shown in our previous study (Bodin et al., 2016), accelerated insulitis was not associated with accelerated diabetes development, suggesting hormesis, *i.e.*, nonmonotonic dose-response resulting potentially in a protective effect at low doses of exposure. Similar results have been seen in epidemiological studies (Conway et al., 2016), however, the mechanism is not yet well understood. In the current study, the lipid changes associated with the number of MPs showed an opposite direction compared with the associations between lipid changes, PFUnDA, insulitis grade and Aac3P cells. The altered lipid metabolism observed in our study may reflect the proinflammatory lipidomic signals of macrophages and is in accordance with changes previously reported as a result of inflammatory activation of MPs (Batista-Gonzalez et al., 2020; Castoldi et al., 2020).

We identified several lipids that were associated with the PFUnDA exposure (30 lipids), insulitis (29 lipids), number of MPs (27/0 lipids, at 7/11 weeks) or Aac-3 s (2/31 lipids, at 7/11 weeks). Specifically, multiple phospholipids, including SMs, PCs and O-PCs were negatively associated with insulitis grade, PFUnDA exposure and Aac3P cells. One specific sphingomyelin, namely SM(d18:1/24:1), showed an opposite pattern than the other SMs, with its levels being increased with increasing PFUnDA exposure levels, and being positively associated with the number of Aac3Ps while showing negative association with the number of MPs. The partial correlation network analysis suggested that these associations were driven by the MPs at week 11. Interestingly, in a recent study, this specific SM was shown to be up-regulated in macrophages isolated from NOD mice treated with IFN-γ + LPS, while the macrophages isolated from a diabetes–resistant mouse strain did not show such an increase (Nelson et al., 2020). The same study further compared NOD mice with a modified NOD mouse strain (NOD- *PLA2G6* ^+/−^ ), the latter type showing substantially less incidence of T1D. The unmodified NOD mice showed significantly lower levels of several SMs, except for SM(d18:1/24:1), in prediabetic stage, suggesting that the SMs are related to Ca^2+^-independent phospholipase A_2_β (iPLA_2_β) activation. As the MP levels in the current study were decreased due to PFUnDA exposure, as was the SM(d18:1/24:1), our results suggest that thespecific SMs measured in circulation may indeed be derived from the pancreatic MPs, and further, potentially reflect the reduced activation stage of the MPs due to the PFUnDA exposure. A recent study comparing three strains of NOD mice (NOD, NOD-E and NOD-SCID mice, with the latter two strains being protected from T1D) showed that several sphingolipids were altered in the pancreas, and they were one of the classes of lipids that discriminated NOD-SCID from NOD and NOD-E mice, thus being potentially linked with T1D development (Murfitt et al., 2018). At a cellular level, several studies have reported extensive, dynamic changes in lipids upon the activation of MPs, with the accumulation of phospholipids, including SMs, as well as CEs and hexosylceramides (Batista-Gonzalez et al., 2020; Castoldi et al., 2020). Particularly, increased ceramides [Cer(d18:1/24:0), Cer(d18:1/22:0), Cer(d18:1/16:0)] have been detected in pro-inflammatory M1 macrophages but not in anti-inflammatory M2 macrophages (Abuawad et al., 2020). In our study, ceramides showed a positive association with the MPs at the early time point, thus potentially reflecting the pro-inflammatory M1 macrophages.

The lipid changes associated with the PFUnDA exposure were similar to those observed in our other animal models, such as in a humanized-PPARα (hPPARα) mouse model exposed to PFOA (Schlezinger et al., 2021), with decreased levels of SMs and alkylPCs. However, there were also distinct differences between the mice models, as in the current NOD mice, the PCs and PUFA-containing TGs were downregulated while this was not seen in the hPPARα mouse model. This suggests that the changes in these two phospholipid classes may be specific to NOD mice.

Overall, the observed changes in the phospholipids in an experimental setup are in good agreement with changes reported in human studies related to T1D, reporting down-regulation of PC, TGs and plasmenyl- (or ether) phospholipids in subjects that developed T1D, including both changes in overall lipid profiles as well as in the levels of individual lipids (Oresic et al., 2008; La Torre et al., 2013; Oresic et al., 2013; Johnson et al., 2019; Li et al., 2020). Importantly, the lipid changes were similar to those in humans observed in the early stages of disease, prior to overt T1D, i.e., prior to the appearance of the first beta cell autoantibodies, as we have reported previously (McGlinchey et al., 2019). The results also agree with the lipid changes reported to be linked with PFAS exposure, both in human studies and in animal models, showing that PFAS exposure is associated with changes particularly in the phospholipids (Salihovic et al., 2018; McGlinchey et al., 2019; Sinisalu et al., 2020). Moreover, we and others have reported that children who progressed to islet autoantibody positivity, or to T1D later in life, have a distinct lipid profile already in the first months of life, already prior to the onset of islet autoimmunity (Fleisch et al., 2017; Sinisalu et al., 2020) and appearing even as early as at birth (Jin et al., 2020). Specifically, these lipid profiles show decreased phospholipid levels, including sphingolipids. The importance of sphingolipid metabolism in the pathogenesis of T1D has been demonstrated in a genome-wide association study that T1D predisposition was associated with multiple gene polymorphisms involved particularly in sphingolipid metabolism (Chen et al., 2020). Importantly, the study also showed that their results also correlated with the degree of islet autoimmunity in subjects with recent-onset T1D. Modified sphingolipid metabolism was also detected in peripheral blood mononuclear cells from children who later developed T1D (Schillemans et al., 2021). In experimental models, it has been reported that exposure to PFOA and PFOS modifies the immune system, with alteration in antibody and cytokine production (Fenton et al., 2021).

We also observed changes in multiple ether PCs as well as in several TGs, which were also associated with the grade of insulitis. Similar changes have been reported in human studies in relation to not only with T1D but also celiac disease (Behr et al., 2020; Jin et al., 2020), which shares common predisposing alleles in the class II HLA-region with T1D (Beggs et al., 2016; Rosen et al., 2017). In our earlier human study, we observed characteristic changes in the TGs in children who later progressed to CD already before the infants were exposed to dietary gluten, suggesting increased *de novo* lipogenesis of these lipids (Beggs et al., 2016). The ether lipids, on the other hand, have structural role in cell membranes and they have also been considered to function as endogenous antioxidant and suggested to be involved also in cell differentiation and signaling pathways (Lu et al., 2019). The ether lipids may also function as endogenous antigens to activate a subset of innate immune cells, more precisely, invariant natural killer T cells (Das et al., 2017) while a therapeutic potential of iNKT cell antigens against autoimmunity has been reported (Bjork et al., 2011). Therefore, the downregulation of the ether lipids could indicate a compromised response to oxidative stress.

Our study also has some limitations. Firstly, we only used female NOD mice in the exposure study and studied only one type of immune cells, i.e., macrophages. The lipid profiles are known to show sex-specific changes. The majority of female NOD mice develop spontaneous disease also without any exposure and thus, comparison of diabetic versus non-diabetic animals was not feasible in this study set-up. This may also partially explain why the accelerated insulitis was not associated with accelerated diabetes development. The results are correlative in nature and even though we did use four levels of exposure, the concentration range may not be sufficient to fully elucidate the reported non-monotonic response of the PFAS exposure.

## Conclusion

Our results show that PFUnDA exposure in NOD mice during early life alters lipid metabolism, and that lipid metabolism is also linked with pancreatic insulitis grade. Importantly, our lipidomics results corresponds closely with data reported in human studies in early stages of T1D development, both supporting the human data and strengthening causality through the use of an experimental exposure model. This suggests that the lipid changes triggered by PFUnDA exposure are linked with activation of macrophages. Also, these agreements strengthen the NOD mouse model as being appropriate for exposome and lipidomics studies in T1D.

## Data Availability

The original contributions presented in the study are included in the article/Supplementary Material, further inquiries can be directed to the corresponding author.
