# Supplementary material for: Lipidomic Analyses Reveal Modulation of Lipid Metabolism by the PFAS Perfluoroundecanoic Acid (PFUnDA) in Non-Obese Diabetic Mice
Source: Front Genet. 2021 Sep 27;12:721507. doi: 10.3389/fgene.2021.721507 (PMC8502800; doi:10.3389/fgene.2021.721507)
Supplement: Supplementary file 1 [file DataSheet1.PDF]

SUPPLEMENTARY DATA

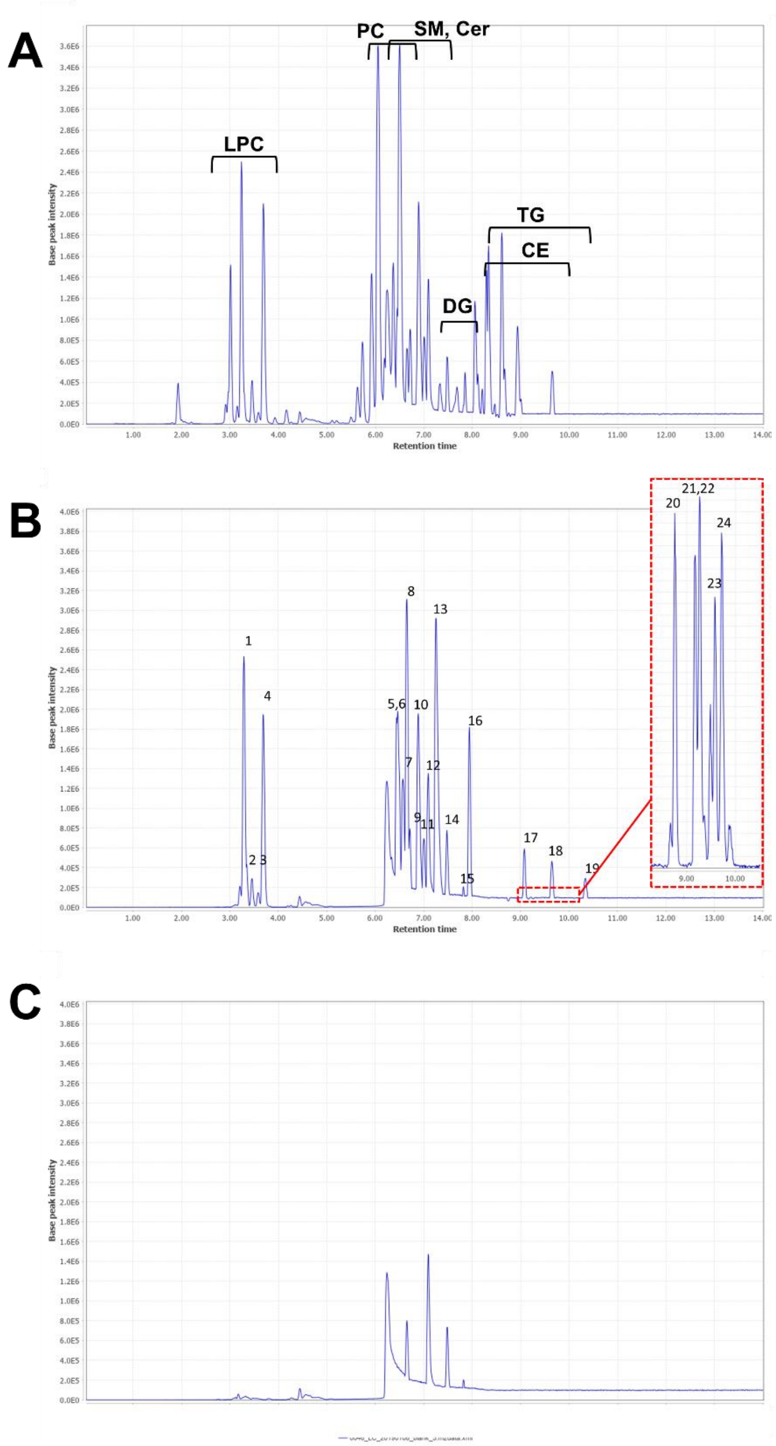

Supplementary Figure 1. Base peak chromatogram of A) pooled sample, B) lipid standard mixture and C) extracted blank sample. Numbering of peaks**:** 1 LysoPE(18:1), 2 LPC(18:1), 3 LPC(17:0), 4 LPC(18:0), 5 SM(d16:1/18:1), 6 PC(16:0/d30/18:1), 7 SM(d18:1/17:0), 8 PC(16:0/18:1), 9 PC(16:0/16:0), 10 PE(16:0/18:1), 11 Cer(d18:1/17:0), 12 PC(17:0/17:0), 13 Cer(d18:1/24:0), 14 PE(17:0/17:0), 15 DG(18:1/18:1)), 16 PC(18:0/18:0), 17 TG(16:0/16:0/16:0), 18 TG(17:0/17:0/17:0), 19 TG(18:0/18:0/18:0), 20 CE(18:1), 21 CE(16:0), 22 CE(18:2) an 22 CE(17:0).

Supplementary Table 1. Calibration curves for lipidomics

	Equation	R2
CE(16:0)	y = 54.482x	R^2 = 0.9831
CE(18:1)	y = 61.47x	R^2 = 0.9976
CE(18:2)	y = 28.072x	R^2 = 0.9871
Cer(d18:1/24:0)	y = 494.95x	R^2 = 0.997
DG(18:1/18:1))	y = 65.274x	R^2 = 0.9958
LPC(18:0)	y = 1545.8x	R^2 = 0.9967
LPC(18:1)	y = 1933.3x	R^2 = 0.9978
LysoPE(18:1)	y = 290.71x	R^2 = 0.9929
PC(16:0/16:0)	y = 1625.9x	R^2 = 0.9981
PC(18:0/18:0)	y = 2753.9x	R^2 = 0.9993
PE(16:0/18:1)	y = 1008.4x	R^2 = 0.9983
TG(16:0/16:0/16:0)	y = 182.46x	R^2 = 0.9991
TG(18:0/18:0/18:0)	y = 119.47x	R^2 = 0.9971

Supplementary table 2. Internal standards, identified lipids and their relative standard deviation in the quality control samples (NIST, in house pooled plasma, pooled extract).

**Lipid**	RSD% NIST	RSD% QC	RSD% Pooled
ISTD_ SM(d18:1/17:0)	14.59	13.62	5.63
ISTD_PC(17:0/17:0)	22.10	20.05	7.88
ISTD_LPC(17:0)	10.63	8.64	4.86
ISTD_LPC(17:0)	11.89	12.23	2.17
ISTD_PC(16:0/d30/18:1)	4.76	6.72	3.22
ISTD_TG(17:0/17:0/17:0)	15.45	15.86	7.15
ISTD_CE(17:0)	26.50	24.28	10.06
ISTD_Cer(d18:1/17:0)	38.78	39.71	13.75
CE(16:0)	110.14	85.89	57.63
CE(16:0)	5.62	4.38	21.07
CE(18:1)	15.28	22.70	9.00
CE(18:2)	19.90	17.77	8.95
CE(20:3)	14.54	14.35	6.92
CE(20:4)	10.93	7.88	12.04
Cer(d18:1/18:1)	34.33	72.09	7.52
Cer(d18:1/24:0)	39.27	43.10	7.65
DG(18:1/18:1))	40.04	28.25	29.72
LPC(16:1)	20.09	4.96	10.95
LPC(18:0)	15.86	4.51	6.83

LPC(18:1)	18.57	4.71	8.10
LPC(18:2)	16.36	5.50	5.92
LPC(20:3)	19.68	6.57	14.97
LPC(20:4)	15.50	6.43	8.29
LPC(22:6)	18.99	8.84	4.68
LysoPE(18:1)	21.99	21.77	8.02
LysoPE(18:1)	12.89	19.34	4.92
PC(16:0/16:0)	14.23	4.67	2.31
PC(16:0e/18:1(9Z))	25.67	14.24	4.22
PC(18:0/18:0)	30.28	26.46	30.07
PC(32:1)	14.58	9.15	14.52
PC(34:1)	14.11	3.74	10.43
PC(34:2)	8.98	3.48	3.98
PC(34:3)	11.88	14.24	5.53
PC(34:3)	60.04	8.40	3.99
PC(34:3)	61.53	11.26	9.10
PC(35:1)	31.73	31.26	8.32
PC(35:2)	13.10	10.52	58.44
PC(35:2)	16.96	5.78	13.83
PC(36:2)	17.78	6.80	15.33
PC(36:2)	46.28	65.26	14.88
PC(36:2)	14.95	4.55	7.04
PC(36:3)	7.63	4.57	1.00
PC(36:3)	14.45	5.96	8.76
PC(36:4)	15.76	11.67	10.70
PC(36:4)	15.91	7.14	1.12
PC(36:4)	11.21	7.82	3.70
PC(36:4)	12.66	9.49	19.25
PC(37:2)	30.86	16.18	19.92
PC(37:4)	47.02	50.26	31.33
PC(37:4)	22.82	31.52	15.47
PC(38:3)	24.91	12.98	87.67
PC(38:3)	28.86	14.85	2.87
PC(38:3)	77.46	24.84	35.84
PC(38:4)	17.08	7.69	30.62
PC(38:4)	16.52	5.70	3.05
PC(38:5)	12.70	6.50	4.51
PC(38:5)	23.81	5.97	30.39
PC(38:6)	16.14	13.42	10.08
PC(38:6)	15.56	9.01	15.31
PC(40:4)	35.83	97.55	18.31
PC(40:4)	33.19	23.60	7.93
PC(40:4)	78.43	40.50	14.12
PC(40:5)	18.88	8.26	22.84
PC(40:5)	18.76	10.28	24.87

PC(40:6)	17.22	5.19	19.00
PC(40:7)	23.33	14.93	38.53
PC(40:7)	17.55	9.92	9.19
PC(40:7)	34.98	17.93	29.46
PC(40:8)	14.32	11.08	13.40
PC(O-34:2)	14.47	9.54	5.58
PC(O-36:4)	11.65	6.49	3.19
PC(O-36:4)	106.35	122.69	6.75
PC(O-36:5)	12.75	7.19	2.03
PC(O-38:4)	14.62	12.97	58.12
PC(O-38:4)	57.56	30.39	66.34
PC(O-38:4)	28.02	18.98	12.17
PC(O-38:5)	16.79	7.19	3.92
PE(16:0/18:1)	64.31	51.10	4.84
PE(O-16:0/22:6) or PE(P-18:0/20:5)	35.89	38.37	65.36
PE(O-16:0/22:6) or PE(P-18:0/20:5)	38.21	23.03	70.24
PE(O-16:0/22:6) or PE(P-18:0/20:5)	114.52	44.71	37.28
PE(O-38:5) or PE(P-38:4)	67.17	69.48	62.32
PE(O-38:5) or PE(P-38:4)	63.95	56.70	19.49
PI(18:0/20:4)	17.46	5.77	6.56
SM(d16:1/18:1) or SM(d18:2/16:0)	20.14	15.61	5.79
SM(d18:1/24:0)	14.18	10.73	1.95
SM(d33:1)	16.53	8.42	5.02
SM(d34:1)	11.68	184.24	128.83
SM(d34:1)	11.75	8.46	2.06
SM(d34:1)	93.60	47.47	65.79
SM(d36:1)	27.29	17.88	12.88
SM(d41:1)	17.01	5.87	8.32
TG(14:0/16:0/18:1)	16.22	7.53	5.69
TG(14:0/18:1/18:1)	15.23	9.86	8.25
TG(14:0/18:2/18:2)	18.47	10.33	17.41
TG(14:0/18:2/18:2)	17.55	9.18	35.05
TG(16:0/16:0/16:0)	12.56	4.45	7.90
TG(16:0/18:0/18:1)	13.17	7.98	18.82
TG(16:0/18:2/18:2)	16.69	9.40	21.49
TG(16:0/18:2/18:2)	94.32	9.22	144.26
TG(16:0/18:2/18:3)	58.65	69.37	44.82
TG(16:0/18:2/18:3)	18.15	11.28	67.72
TG(16:0/22:5/18:1) or TG(20:4/18:1/18:1)	16.68	11.02	41.30
TG(16:0/22:5/18:1) or TG(20:4/18:1/18:1)	15.19	5.83	35.05
TG(16:0/22:5/18:1) or TG(20:4/18:1/18:1)	78.60	89.38	33.03
TG(18:0/18:0/18:0)	100.55	67.18	48.24

TG(18:0/18:1/20:4)	83.60	99.10	30.34
TG(18:0/18:1/20:4)	17.08	29.53	16.78
TG(18:0/18:1/20:4)	17.61	12.69	6.70
TG(18:1/12:0/18:1) or TG(18:2/16:0/14:0)	16.93	8.56	3.77
TG(18:1/18:1/16:0)	15.08	12.36	6.83
TG(18:1/18:1/18:1)	21.31	12.86	11.39
TG(18:1/18:1/22:6)	64.38	85.44	6.43
TG(18:1/18:1/22:6)	22.38	14.18	59.45
TG(18:1/18:1/22:6)	22.23	7.25	35.20
TG(18:1/18:2/18:2)	18.38	11.06	17.45
TG(18:1/18:2/18:2)	16.45	11.38	10.77
TG(18:2/18:1/16:0)	15.17	11.55	14.33
TG(18:2/18:1/18:1)	18.68	12.84	14.33
TG(18:2/18:1/18:1)	35.11	37.95	42.76
TG(18:2/18:2/18:2) or TG(18:3/18:2/18:1)	20.42	15.92	25.41
TG(18:2/18:2/18:2) or TG(18:3/18:2/18:1)	18.05	8.00	22.28
TG(18:2/22:5/16:0)	19.98	8.39	43.11
TG(18:2/22:5/16:0)	13.08	9.03	32.65
TG(50:0)	28.07	16.98	22.86
TG(50:2)	18.10	11.82	6.22
TG(50:3)	16.05	8.58	7.81
TG(50:5)	45.90	44.77	42.38
TG(50:5)	40.37	55.39	23.99
TG(50:5)	25.45	65.43	37.45
TG(51:2)	20.53	13.97	18.78
TG(51:3)	16.43	9.50	22.97
TG(51:4)	18.58	10.73	29.78
TG(52:2)	15.59	6.96	7.42
TG(52:3)	9.38	5.33	6.83
TG(52:4)	21.32	18.82	12.89
TG(52:4)	156.06	17.73	33.05
TG(52:5)	39.89	45.57	19.27
TG(52:6)	7.16	48.93	39.61
TG(52:6)	44.70	93.88	36.94
TG(52:6)	75.74	121.21	38.50
TG(53:3)	17.15	9.33	19.23
TG(53:4)	66.92	16.60	25.93
TG(53:4)	13.83	7.61	27.51
TG(53:5)	57.35	45.53	41.70
TG(54:1)	20.50	12.60	14.31
TG(54:1)	14.80	6.10	11.26
TG(54:2)	19.87	12.67	19.07

TG(54:3)	22.53	14.97	6.37
TG(54:4)	13.93	15.00	8.09
TG(54:4)	61.76	53.04	31.23
TG(54:5)	24.25	20.93	7.70
TG(54:5)	26.06	24.62	6.66
TG(54:6)	36.87	35.04	13.16
TG(54:6)	39.29	36.03	4.64
TG(54:6)	122.67	10.28	27.56
TG(54:7)	47.97	66.45	38.23
TG(54:7)	47.38	31.10	35.41
TG(54:7)	73.80	40.05	37.80
TG(54:7)	22.25	8.92	28.68
TG(56:2)	26.60	6.35	16.37
TG(56:2)	51.74	52.31	25.03
TG(56:4)	23.73	15.62	17.95
TG(56:5)	54.27	91.25	20.18
TG(56:8)	20.39	13.17	30.52
TG(56:8)	9.84	9.97	36.89
TG(58:9)	63.68	75.56	77.40
TG(58:9)	17.70	59.59	83.52
TG(58:9)	20.01	13.35	29.44
